# Comparative Evaluation of Photodynamic Therapy and Photoablation as Adjuncts to Open Flap Debridement Versus Open Flap Debridement Alone in Periodontitis Patients: A Double-Blinded Randomised Controlled Trial

**DOI:** 10.7759/cureus.114055

**Published:** 2026-08-06

**Authors:** Raghavendra Metri, Snehal Jadhav, Medara Ramakanth, Pradumna Niturkar, Rohini Bhise, Akanksha Jadhav

**Affiliations:** 1 Department of Periodontology, Maharashtra Institute of Dental Sciences and Research, Dental College, Latur, IND; 2 Department of Periodontology and Implantology, Sai Chandra Dentistry, Lingampally, IND

**Keywords:** chronic periodontitis, diode laser, open flap debridement, photoablation, photodynamic therapy

## Abstract

Background: Residual periodontal pockets may persist after Phase I therapy, contributing to recurrent inflammation and progressive periodontal breakdown. Photodynamic therapy (PDT) and photoablation have been proposed as adjuncts to open flap debridement, although direct comparative evidence remains limited.

Objective: This study aims to compare the clinical effectiveness of PDT and photoablation as adjuncts to open flap debridement with that of open flap debridement alone in patients with chronic periodontitis.

Methods: This participant- and outcome-assessor-blinded, parallel-arm randomised controlled trial enrolled 51 patients with residual probing pocket depths of 5-7 mm after Phase I therapy. Seventeen participants were allocated to each of three groups: open flap debridement alone, open flap debridement with PDT using methylene blue and a 650 nm diode laser, or open flap debridement with photoablation using a 980 nm diode laser. Forty-five participants completed the six-month follow-up. Plaque Index, Gingival Index, Probing Pocket Depth, Clinical Attachment Level, Gingival Recession, and Papillary Bleeding Index were assessed at baseline, three months, and six months.

Results: All groups improved significantly over time. At six months, Clinical Attachment Level, Gingival Recession, and Papillary Bleeding Index differed significantly among the groups (all p < 0.001). Mean Clinical Attachment Level values were 3.93 ± 1.10 mm, 2.47 ± 0.74 mm, and 2.80 ± 0.56 mm in the control, PDT, and photoablation groups, respectively. Corresponding Gingival Recession values were 1.60 ± 1.06 mm, 0.27 ± 0.46 mm, and 0.53 ± 0.52 mm, while Papillary Bleeding Index values were 0.59 ± 0.12, 0.41 ± 0.07, and 0.45 ± 0.07. Plaque Index, Gingival Index, and Probing Pocket Depth remained comparable among the groups. No significant differences were observed between the two laser-assisted groups.

Conclusion: PDT and photoablation provided comparable additional benefits when combined with open flap debridement, particularly for clinical attachment, gingival recession, and bleeding control. Larger multicentre trials with longer follow-up are required to confirm the durability and clinical applicability of these findings.

## Introduction

Periodontitis is a chronic inflammatory condition characterised by progressive destruction of the periodontal ligament, cementum, and alveolar bone [1]. It results from persistent microbial challenge combined with a dysregulated host-mediated inflammatory response within the periodontal tissues. Conventional mechanical therapy may not completely eliminate inflammation because microorganisms can persist within structured biofilms, periodontal pockets, pocket epithelium, connective tissues, and irregular root-surface anatomy. Persistent microorganisms may contribute to recurrent inflammation, delayed healing, and further periodontal attachment loss [1]. This persistence is closely linked to microbial dysbiosis, in which the periodontal microbial community shifts from health-associated commensals towards a disease-promoting biofilm that sustains inflammation and tissue destruction [2]. Microbial control remains a central objective of periodontal therapy because dysbiosis can alter local immune responses, promote inflammatory mediator release, and contribute to periodontal tissue destruction [2].

Mechanical periodontal therapy remains the cornerstone of periodontal management [3]. Effective removal of plaque and calculus reduces periodontal inflammation, and repeated subgingival instrumentation can improve pocket closure in stage III-IV periodontitis [3]. Within this non-surgical approach, scaling and root planing serve as the principal minimally invasive procedures for disrupting subgingival biofilm, removing calculus deposits, and creating a biologically acceptable root surface [4]. Despite these benefits, complete debridement may remain difficult in deep periodontal pockets, furcation areas, root concavities, and other anatomically complex sites [4]. Residual periodontal pockets may therefore persist after Phase I therapy and require surgical access through open flap debridement [3,4].

Open flap debridement provides direct visualisation of root surfaces and periodontal defects, permitting more complete removal of subgingival deposits and inflamed granulation tissue [3,4]. Despite improved surgical access, the procedure may not completely eliminate devitalised tissue, diseased pocket epithelium, or microorganisms located within root-surface irregularities and soft-tissue walls [1,2]. Adjunctive treatment approaches have consequently been investigated to enhance antimicrobial activity and improve clinical outcomes in periodontal and peri-implant inflammatory diseases [5]. Evidence from adjunctive protocols combined with mechanical debridement suggests that antimicrobial or bioactive measures may complement conventional periodontal therapy in selected clinical situations [5]. Beyond directly targeting microbial burden and local tissue responses, adjunctive strategies have also expanded towards host-microbial modulation, including approaches designed to modify mucosal immune responses to major periodontal pathogens such as *Porphyromonas gingivalis* [6].

Photodynamic therapy (PDT) is a biologically plausible adjunctive approach in periodontal treatment. It involves the application of a photosensitising agent followed by light activation to generate antimicrobial and cellular effects relevant to periodontal healing [7]. When used specifically to reduce periodontal microbial burden, this approach is commonly described as antimicrobial PDT [8]. Clinical evidence indicates that antimicrobial PDT can improve the management of residual periodontal pockets during maintenance therapy [9,10]. Indocyanine green-based antimicrobial PDT has also produced clinical and microbiological benefits when used as an adjunct to non-surgical periodontal treatment [11]. A one-year randomised controlled trial further reported clinical, microbiological, and immunological outcomes following antimicrobial PDT for recurrent or persistent periodontal pockets [12]. PDT may also enhance cellular activity on periodontal regenerative materials and thereby support tissue repair and wound healing [7]. Other adjunctive approaches, including probiotic protocols combined with non-surgical periodontal therapy, reflect the broader development of multimodal periodontal management [8]. These strategies aim to complement mechanical debridement by targeting biofilm ecology, inflammation, and periodontal tissue responses [8]. Beyond PDT, various other laser-based periodontal techniques have also been evaluated as adjuncts to periodontal pocket treatment [10]. Dual-wavelength diode laser therapy has shown clinical efficacy when combined with non-surgical periodontal treatment, supporting the broader therapeutic potential of laser-assisted protocols in periodontitis [10].

Despite these findings, clinically relevant evidence gaps remain. Available studies have predominantly focused on non-surgical periodontal treatment, maintenance therapy, peri-implant disease, or PDT as an isolated adjunctive intervention [5,9-12]. Direct comparative evidence on PDT and photoablation used with open flap debridement remains limited [12]. Photoablation may provide benefit through the removal of diseased pocket epithelium and inflamed soft tissue, whereas PDT primarily exerts antimicrobial effects through light-mediated activation of a photosensitising agent [7,10]. It remains uncertain whether these two laser-assisted approaches provide comparable or superior clinical outcomes relative to open flap debridement alone [9-12]. The present study, therefore, compared PDT and photoablation as separate adjuncts to open flap debridement with open flap debridement alone in patients with chronic periodontitis.

Objectives of the study

The objective of this study was to evaluate and compare the clinical effectiveness of PDT and photoablation used as adjuncts to open flap debridement in patients with chronic periodontitis. Changes in Plaque Index, Gingival Index, Probing Pocket Depth, Clinical Attachment Level, Gingival Recession, and Papillary Bleeding Index were assessed at baseline, three months, and six months. The study also examined whether either laser-assisted adjunct produced superior clinical outcomes compared with open flap debridement alone.

## Materials and methods

Study design

The present study was designed as a participant- and outcome-assessor-blinded, parallel-arm, randomised controlled clinical trial to compare the clinical effectiveness of PDT and photoablation as adjuncts to open flap debridement with open flap debridement alone in patients with chronic periodontitis. The trial included three intervention groups with a 1:1:1 allocation ratio and clinical assessments at baseline, three months, and six months.

Blinding

Participants and the clinical examiner responsible for recording periodontal parameters were blinded to group allocation. The operator performing the surgical and laser procedures could not be blinded because of the distinct nature of the interventions. Treatment allocation was not disclosed to the blinded examiner during clinical outcome assessment.

Study setting and ethical approval

The study was conducted at the Department of Periodontology, Maharashtra Institute of Dental Sciences & Research (MIDSR) Dental College, Latur. Ethical approval was obtained from the Institutional Ethics Committee of the MIDSR Centre, Latur. The approval letter was issued for the protocol titled “Comparative Evaluation of the Effect of Photodynamic Therapy and Photoablation as an Adjunct to Open Flap Debridement to Open Flap Debridement Alone in Periodontitis Patients: A Double-Blinded Randomised Controlled Trial.” The Institutional Ethics Committee recommended the study for approval under registration/proposal number MIDSR/1518/517/IEC-189/2024, dated April 18, 2024. 

The trial was prospectively registered with the Clinical Trials Registry-India under registration number CTRI/2024/08/072248 on August 9, 2024. The registered primary outcomes were Probing Pocket Depth and Clinical Attachment Level, whereas Plaque Index, Gingival Index, and bleeding assessment were registered as secondary outcomes. Papillary Bleeding Index was used as the clinical measure of bleeding, and Gingival Recession was evaluated as an additional exploratory clinical parameter. Written informed consent was obtained from all participants before enrollment. The study was conducted in accordance with the approved protocol and applicable ethical principles for clinical research involving human participants.

Study population

Patients diagnosed with chronic periodontitis who visited the Department of Periodontology and required periodontal flap surgery were screened for eligibility. Patients with residual periodontal pockets after completion of Phase I periodontal therapy were considered for participation. Eligibility was determined after clinical examination and confirmation that residual pocketing persisted despite completion of initial periodontal treatment.

Sample size calculation

The sample size was calculated using OpenEpi software (version 3.04, OpenEpi Development Team, Atlanta, Georgia), with Clinical Attachment Level selected as the primary outcome for the calculation. The anticipated mean difference and standard deviation were derived from the randomised clinical trial by Kolamala et al. [13], which compared diode laser-assisted open flap debridement with conventional open flap debridement. The calculation was performed using a 95% confidence interval, 80% study power, 5% level of significance, and a 1:1:1 allocation ratio among the three study groups.

The minimum required sample size was 13 participants per group. After accounting for an anticipated 15% attrition rate, the required sample increased to 15 participants per group. Although the registered target sample size was 45, 51 participants were ultimately enrolled and randomised, with 17 participants assigned to each group, to ensure that at least 15 participants per group completed follow-up. Forty-five participants completed the six-month follow-up and were included in the final analysis.

Inclusion criteria

Patients were included if they were aged between 18 and 55 years, diagnosed with chronic periodontitis, and had a residual Probing Pocket Depth of 5-7 mm after Phase I periodontal therapy. Only patients classified as American Society of Anesthesiologists physical status I or II were included. Patients willing to comply with the study protocol and follow-up visits and those who provided written informed consent were enrolled.

Exclusion criteria

Patients were excluded if they had poor oral hygiene, defined as a Plaque Index ≥1 on the follow-up day after Phase I therapy. Patients with teeth exhibiting greater than grade II mobility or grade III or IV furcation involvement were also excluded. Smokers, tobacco users, pregnant or lactating women, patients who had received antibiotic therapy within the preceding three months, patients who had undergone periodontal surgery within the previous six months, and patients with a known allergy to methylene blue dye were excluded from the study.

Randomisation and allocation concealment

Eligible participants were randomly assigned to the three study groups using a computer-generated randomisation sequence. Allocation concealment was maintained using sequentially numbered, opaque, sealed envelopes, which were opened at the time of intervention. The allocation sequence remained concealed until the participant had been enrolled and was ready to receive the assigned intervention.

Group A received open flap debridement alone. Group B received open flap debridement with adjunctive PDT using methylene blue dye and a 650 nm diode laser. Group C received open flap debridement with adjunctive photoablation therapy using a 980 nm diode laser.

Clinical parameters and outcome assessment

Clinical parameters were recorded at baseline, three months, and six months. The evaluated parameters included Plaque Index, Gingival Index, Probing Pocket Depth, Clinical Attachment Level, Gingival Recession, and Papillary Bleeding Index. Probing Pocket Depth and Clinical Attachment Level constituted the registered primary outcomes. Plaque Index, Gingival Index, and Papillary Bleeding Index were secondary outcomes, while Gingival Recession was analysed as an exploratory outcome.

All measurements were recorded by the same blinded examiner to reduce assessment bias. A standardised clinical measurement protocol was followed consistently at all assessment visits. Formal examiner calibration and quantitative assessment of intra-examiner reliability were not performed; therefore, kappa coefficients or intraclass correlation coefficients were not available.

Surgical procedures

Group A: Open Flap Debridement

After administration of local anaesthesia using 2% lignocaine with adrenaline at a concentration of 1:80,000, open flap debridement was performed. Full-thickness mucoperiosteal flaps were reflected to provide access to the periodontal defect areas. Thorough debridement and root planing were performed, and inflamed granulation tissue was removed. The flaps were then repositioned and sutured using 4-0 silk sutures.

Group B: Open Flap Debridement With PDT

After completion of open flap debridement, methylene blue dye was applied to the surgical site as the photosensitising agent. The dye was allowed to remain at the site for three minutes before irradiation. Laser irradiation was then performed using a 650 nm diode laser with a 400 µm tip in continuous contact mode. The laser was applied at a power output of 0.2 W for 10 seconds at each treated site. The intervention was intended to provide light-activated antimicrobial treatment after mechanical and surgical debridement.

Group C: Open Flap Debridement With Photoablation Therapy

After completion of open flap debridement, photoablation was performed using a 980 nm diode laser. The laser was applied to the inner epithelial lining of the periodontal flap to remove inflamed epithelial tissue. Irradiation was carried out using a 400 µm tip in continuous contact mode at a power output of 3 W for 10 seconds at each treated site.

Follow-up assessment

Participants were clinically reassessed at three and six months after treatment. The same periodontal outcomes recorded at baseline were evaluated at both follow-up visits by the blinded examiner using the standardised assessment protocol. Participant retention, losses to follow-up, and the number included in the final analysis were documented using a CONSORT (Consolidated Standards of Reporting Trials) flowchart.

Statistical analysis

Data were entered into Microsoft Excel (Microsoft Corporation, Redmond, Washington) and analysed using IBM SPSS Statistics for Windows, Version 22 (Released 2013; IBM Corp., Armonk, New York). The normality of data distribution was assessed using the Kolmogorov-Smirnov test. Plaque Index, Probing Pocket Depth, Clinical Attachment Level, and Gingival Recession showed non-normal distributions in all three groups. Gingival Index showed a non-normal distribution in the control and photoablation groups but a normal distribution in the PDT group. Papillary Bleeding Index showed a normal distribution in the control and PDT groups and a non-normal distribution in the photoablation group.

Intragroup comparisons across baseline, three months, and six months were performed using Friedman’s test for non-normally distributed variables and repeated-measures analysis of variance for normally distributed variables. Friedman’s test results were reported as χ² values with two degrees of freedom, whereas repeated-measures analysis of variance results were reported as F-values with numerator and denominator degrees of freedom. Bonferroni correction was applied to applicable post hoc pairwise comparisons.

Intergroup comparisons among the three treatment groups at each time point were performed using the Kruskal-Wallis test. Kruskal-Wallis results were reported as H values with two degrees of freedom. When statistically significant overall differences were observed, Bonferroni-adjusted post hoc pairwise comparisons were applied.

Effect sizes were reported in addition to p-values. Kendall’s W was used for Friedman’s tests, partial eta squared (partial η²) was used for repeated-measures analysis of variance, and eta squared based on the Kruskal-Wallis H statistic (ηH²) was used for intergroup comparisons. Where only threshold test statistics were available, effect sizes were reported as minimum or lower-bound estimates.

Data were expressed as mean ± standard deviation. Categorical participant data were reported as numbers and percentages (n (%)). The primary analysis was a complete-case analysis restricted to the 45 participants who completed the six-month follow-up; no imputation of missing follow-up data was performed. A p-value <0.05 was considered statistically significant.

## Results

The study included 51 patients aged 35-54 years, with a mean age of 44.5 ± 5.49 years. Among them, 27 (52.94%) were female and 24 (47.06%) were male. Seventeen participants were allocated to each treatment group. The three groups were comparable for all baseline clinical parameters, with no statistically significant intergroup differences. Baseline demographic and clinical characteristics are presented in Table 1.

**Table 1:** Baseline demographic and clinical characteristics

Characteristics	Overall Participants (n=51)
Age, years, mean ± SD	44.5 ± 5.49
Age range, years	35-54
Female sex, n (%)	27 (52.94)
Male sex, n (%)	24 (47.06)

Baseline clinical characteristics were comparable across the control, PDT, and photoablation groups, with no statistically significant intergroup differences (Table 2).

**Table 2:** Baseline clinical characteristics by treatment group Data are presented as mean ± standard deviation or number (percentage). Baseline clinical parameters were compared using the Kruskal-Wallis test. PDT: photodynamic therapy; PAT: photoablation therapy; SD: standard deviation.

Clinical Parameter	Control (n=17)	PDT (n=17)	PAT (n=17)	p-value
Plaque Index, mean ± SD	0.75 ± 0.10	0.76 ± 0.10	0.77 ± 0.10	0.842
Gingival Index, mean ± SD	1.57 ± 0.30	1.61 ± 0.32	1.55 ± 0.29	0.864
Probing Pocket Depth, mm, mean ± SD	6.67 ± 0.49	6.73 ± 0.46	6.80 ± 0.41	0.717
Clinical Attachment Level, mm, mean ± SD	5.07 ± 0.80	4.73 ± 0.70	4.93 ± 0.80	0.497
Gingival Recession, mm, mean ± SD	−1.60 ± 0.74	−2.00 ± 0.93	−1.87 ± 0.74	0.313
Papillary Bleeding Index, mean ± SD	2.23 ± 0.41	2.05 ± 0.35	2.02 ± 0.44	0.306

**Figure 1:**
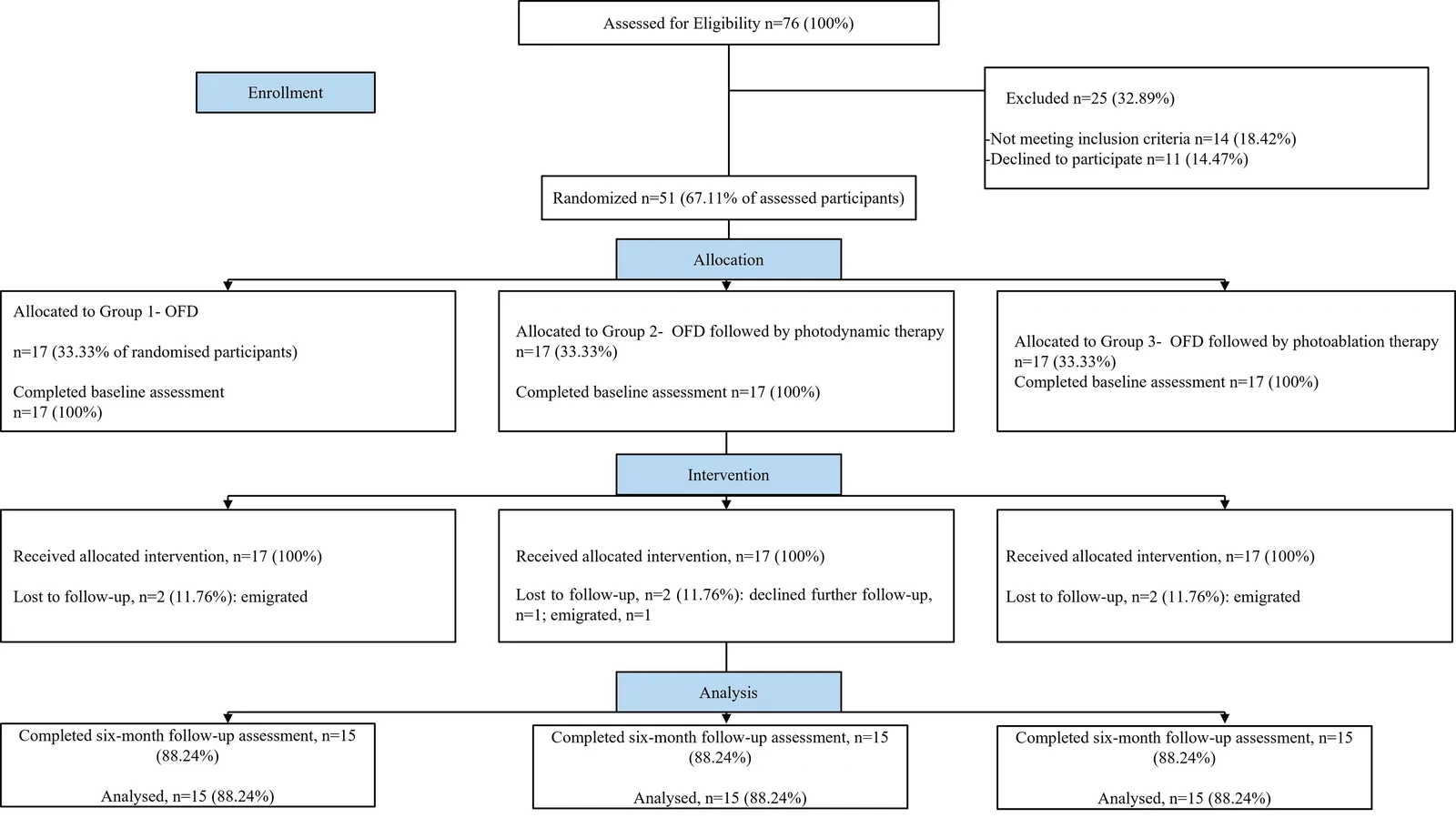
CONSORT flowchart showing participant enrollment, allocation, follow-up, and analysis The figure shows participant data as numbers and percentages, n (%). Percentages for screening and exclusion were calculated using the 76 assessed participants, allocation percentages were calculated using the 51 randomised participants, and follow-up percentages were calculated using the 17 participants allocated to each treatment group. CONSORT: Consolidated Standards of Reporting Trials, OFD: open flap debridement.

Participant enrollment, allocation, follow-up, and analysis are shown in Figure 1.

Intragroup comparison

All three groups (n=15 per group) showed significant clinical improvement from baseline to three and six months. Plaque Index and Gingival Index decreased in the control, PDT, and photoablation therapy groups, indicating improved plaque control and gingival health.

Probing Pocket Depth decreased significantly in all groups. At six months, the mean Probing Pocket Depth was 2.33 ± 0.49 mm in the control group, 2.20 ± 0.68 mm in the PDT group, and 2.20 ± 0.41 mm in the photoablation therapy group.

Clinical Attachment Level also improved in all groups, with greater improvement observed in the laser-assisted groups. At six months, the mean Clinical Attachment Level was 3.93 ± 1.10 mm in the control group, 2.47 ± 0.74 mm in the PDT group, and 2.80 ± 0.56 mm in the photoablation therapy group.

Gingival Recession and Papillary Bleeding Index showed significant changes during follow-up. At six months, gingival recession values were lower in the PDT and photoablation therapy groups than in the control group. Papillary Bleeding Index decreased from 2.23 ± 0.41 to 0.59 ± 0.12 in the control group, from 2.05 ± 0.35 to 0.41 ± 0.07 in the PDT group, and from 2.02 ± 0.44 to 0.45 ± 0.07 in the photoablation therapy group. These continuous clinical variables are presented as mean ± standard deviation; percentages are not applicable to these measurements. Intragroup clinical changes are shown in Table 3.

**Table 3:** Intragroup comparison of clinical parameters Effect sizes are reported as Kendall’s W for Friedman’s test and partial eta squared (partial η²) for repeated-measures ANOVA. Because the available statistical output reported threshold rather than exact test-statistic values, the effect sizes are presented as minimum estimates. Values of Kendall’s W above 0.461 and partial η² above 0.389 indicate large within-group effects. A p-value <0.05 was considered statistically significant. * indicates statistical significance. PI: Plaque Index; GI: Gingival Index; PPD: Probing Pocket Depth; CAL: Clinical Attachment Level; REC: Gingival Recession; PBI: Papillary Bleeding Index; PDT: photodynamic therapy; PAT: photoablation therapy; SD: standard deviation; ANOVA: analysis of variance.

Parameter	Group	Baseline (Mean ± SD)	3 Months (Mean ± SD)	6 Months (Mean ± SD)	Statistical Test	Test Statistic	Effect Size	p-value
PI	Control	0.75 ± 0.10	0.53 ± 0.13	0.59 ± 0.15	Friedman’s test	χ²(2) ≥ 13.82	Kendall’s W ≥ 0.461	0.001*
PI	PDT	0.76 ± 0.10	0.53 ± 0.11	0.52 ± 0.09	Friedman’s test	χ²(2) > 13.82	Kendall’s W > 0.461	<0.001*
PI	PAT	0.77 ± 0.10	0.57 ± 0.13	0.53 ± 0.13	Friedman’s test	χ²(2) > 13.82	Kendall’s W > 0.461	<0.001*
GI	Control	1.57 ± 0.30	0.77 ± 0.10	0.76 ± 0.09	Friedman’s test	χ²(2) > 13.82	Kendall’s W > 0.461	<0.001*
GI	PDT	1.61 ± 0.32	0.79 ± 0.11	0.74 ± 0.09	Repeated-measures ANOVA	F(2,28) > 8.93	Partial η² > 0.389	<0.001*
GI	PAT	1.55 ± 0.29	0.78 ± 0.11	0.73 ± 0.08	Friedman’s test	χ²(2) > 13.82	Kendall’s W > 0.461	<0.001*
PPD (mm)	Control	6.67 ± 0.49	3.13 ± 0.64	2.33 ± 0.49	Friedman’s test	χ²(2) > 13.82	Kendall’s W > 0.461	<0.001*
PPD (mm)	PDT	6.73 ± 0.46	3.00 ± 0.54	2.20 ± 0.68	Friedman’s test	χ²(2) > 13.82	Kendall’s W > 0.461	<0.001*
PPD (mm)	PAT	6.80 ± 0.41	2.80 ± 0.56	2.20 ± 0.41	Friedman’s test	χ²(2) > 13.82	Kendall’s W > 0.461	<0.001*
CAL (mm)	Control	5.07 ± 0.80	3.67 ± 0.90	3.93 ± 1.10	Friedman’s test	χ²(2) ≥ 13.82	Kendall’s W ≥ 0.461	0.001*
CAL (mm)	PDT	4.73 ± 0.70	3.40 ± 0.63	2.47 ± 0.74	Friedman’s test	χ²(2) > 13.82	Kendall’s W > 0.461	<0.001*
CAL (mm)	PAT	4.93 ± 0.80	3.27 ± 0.46	2.80 ± 0.56	Friedman’s test	χ²(2) > 13.82	Kendall’s W > 0.461	<0.001*
REC (mm)	Control	−1.60 ± 0.74	0.53 ± 0.64	1.60 ± 1.06	Friedman’s test	χ²(2) > 13.82	Kendall’s W > 0.461	<0.001*
REC (mm)	PDT	−2.00 ± 0.93	0.40 ± 0.51	0.27 ± 0.46	Friedman’s test	χ²(2) > 13.82	Kendall’s W > 0.461	<0.001*
REC (mm)	PAT	−1.87 ± 0.74	0.47 ± 0.64	0.53 ± 0.52	Friedman’s test	χ²(2) > 13.82	Kendall’s W > 0.461	<0.001*
PBI	Control	2.23 ± 0.41	0.88 ± 0.20	0.59 ± 0.12	Repeated-measures ANOVA	F(2,28) > 8.93	Partial η² > 0.389	<0.001*
PBI	PDT	2.05 ± 0.35	0.73 ± 0.21	0.41 ± 0.07	Repeated-measures ANOVA	F(2,28) > 8.93	Partial η² > 0.389	<0.001*
PBI	PAT	2.02 ± 0.44	0.80 ± 0.17	0.45 ± 0.07	Friedman’s test	χ²(2) > 13.82	Kendall’s W > 0.461	<0.001*

At baseline, all clinical parameters were comparable among the three groups. No statistically significant differences were observed in Plaque Index, Gingival Index, Probing Pocket Depth, Clinical Attachment Level, Gingival Recession, or Papillary Bleeding Index, indicating adequate baseline similarity among the treatment groups.

At three months, intergroup comparisons showed no statistically significant differences for any evaluated clinical parameter.

At six months, Plaque Index, Gingival Index, and Probing Pocket Depth remained statistically comparable among the groups. The mean Probing Pocket Depth was 2.33 ± 0.49 mm in the control group, 2.20 ± 0.68 mm in the PDT group, and 2.20 ± 0.41 mm in the photoablation therapy group.

Significant intergroup differences were observed at six months for Clinical Attachment Level, Gingival Recession, and Papillary Bleeding Index (all p<0.001). Clinical Attachment Level was 3.93 ± 1.10 mm in the control group, 2.47 ± 0.74 mm in the PDT group, and 2.80 ± 0.56 mm in the photoablation therapy group. Gingival Recession values were 1.60 ± 1.06 mm, 0.27 ± 0.46 mm, and 0.53 ± 0.52 mm in the control, PDT, and photoablation therapy groups, respectively. Papillary Bleeding Index values were 0.59 ± 0.12, 0.41 ± 0.07, and 0.45 ± 0.07, respectively. All continuous clinical data are presented as mean ± standard deviation, and percentages are not applicable to these outcome measurements. Intergroup clinical comparisons are shown in Table 4.

**Table 4:** Intergroup comparison of clinical parameters with effect sizes Data are presented as mean ± standard deviation (SD), with n=15 participants in each group. Intergroup comparisons were performed using the Kruskal-Wallis test. Effect sizes were calculated as eta squared based on the Kruskal-Wallis H statistic using ηH² = H/(N−1), where N=45. Values of approximately 0.01, 0.06, and 0.14 were interpreted as small, moderate, and large effects, respectively. Because exact H values were unavailable for the significant six-month comparisons, the corresponding effect sizes are reported as lower-bound estimates. A p-value <0.05 was considered statistically significant. * indicates statistical significance. PI: Plaque Index; GI: Gingival Index; PPD: Probing Pocket Depth; CAL: Clinical Attachment Level; REC: Gingival Recession; PBI: Papillary Bleeding Index; PDT: photodynamic therapy; PAT: photoablation therapy; SD: standard deviation; ηH²: eta squared based on the Kruskal-Wallis H statistic.

Parameter	Time Point	Control (Mean ± SD)	PDT (Mean ± SD)	PAT (Mean ± SD)	H Value	Effect Size (ηH²)	p-value
PI	Baseline	0.75 ± 0.10	0.76 ± 0.10	0.77 ± 0.10	H(2)=0.344	0.008	0.842
PI	3 Months	0.53 ± 0.13	0.53 ± 0.11	0.57 ± 0.13	H(2)=0.998	0.023	0.607
PI	6 Months	0.59 ± 0.15	0.52 ± 0.09	0.53 ± 0.13	H(2)=2.128	0.048	0.345
GI	Baseline	1.57 ± 0.30	1.61 ± 0.32	1.55 ± 0.29	H(2)=0.292	0.007	0.864
GI	3 Months	0.77 ± 0.10	0.79 ± 0.11	0.78 ± 0.11	H(2)=0.544	0.012	0.762
GI	6 Months	0.76 ± 0.09	0.74 ± 0.09	0.73 ± 0.08	H(2)=0.976	0.022	0.614
PPD (mm)	Baseline	6.67 ± 0.49	6.73 ± 0.46	6.80 ± 0.41	H(2)=0.665	0.015	0.717
PPD (mm)	3 Months	3.13 ± 0.64	3.00 ± 0.54	2.80 ± 0.56	H(2)=2.469	0.056	0.291
PPD (mm)	6 Months	2.33 ± 0.49	2.20 ± 0.68	2.20 ± 0.41	H(2)=0.567	0.013	0.753
CAL (mm)	Baseline	5.07 ± 0.80	4.73 ± 0.70	4.93 ± 0.80	H(2)=1.398	0.032	0.497
CAL (mm)	3 Months	3.67 ± 0.90	3.40 ± 0.63	3.27 ± 0.46	H(2)=3.301	0.075	0.192
CAL (mm)	6 Months	3.93 ± 1.10	2.47 ± 0.74	2.80 ± 0.56	H(2)>13.816	>0.314	<0.001*
REC (mm)	Baseline	−1.60 ± 0.74	−2.00 ± 0.93	−1.87 ± 0.74	H(2)=2.323	0.053	0.313
REC (mm)	3 Months	0.53 ± 0.64	0.40 ± 0.51	0.47 ± 0.64	H(2)=0.258	0.006	0.879
REC (mm)	6 Months	1.60 ± 1.06	0.27 ± 0.46	0.53 ± 0.52	H(2)>13.816	>0.314	<0.001*
PBI	Baseline	2.23 ± 0.41	2.05 ± 0.35	2.02 ± 0.44	H(2)=2.368	0.054	0.306
PBI	3 Months	0.88 ± 0.20	0.73 ± 0.21	0.80 ± 0.17	H(2)=3.019	0.069	0.221
PBI	6 Months	0.59 ± 0.12	0.41 ± 0.07	0.45 ± 0.07	H(2)>13.816	>0.314	<0.001*

Post hoc analysis showed significantly greater Clinical Attachment Level gain, lower Gingival Recession, and reduced Papillary Bleeding Index in both laser-assisted groups (n=15 per group) compared with open flap debridement alone. No significant difference was observed between PDT and photoablation therapy, indicating comparable clinical effectiveness of the two adjunctive modalities.

Clinical figures

**Figure 2:**
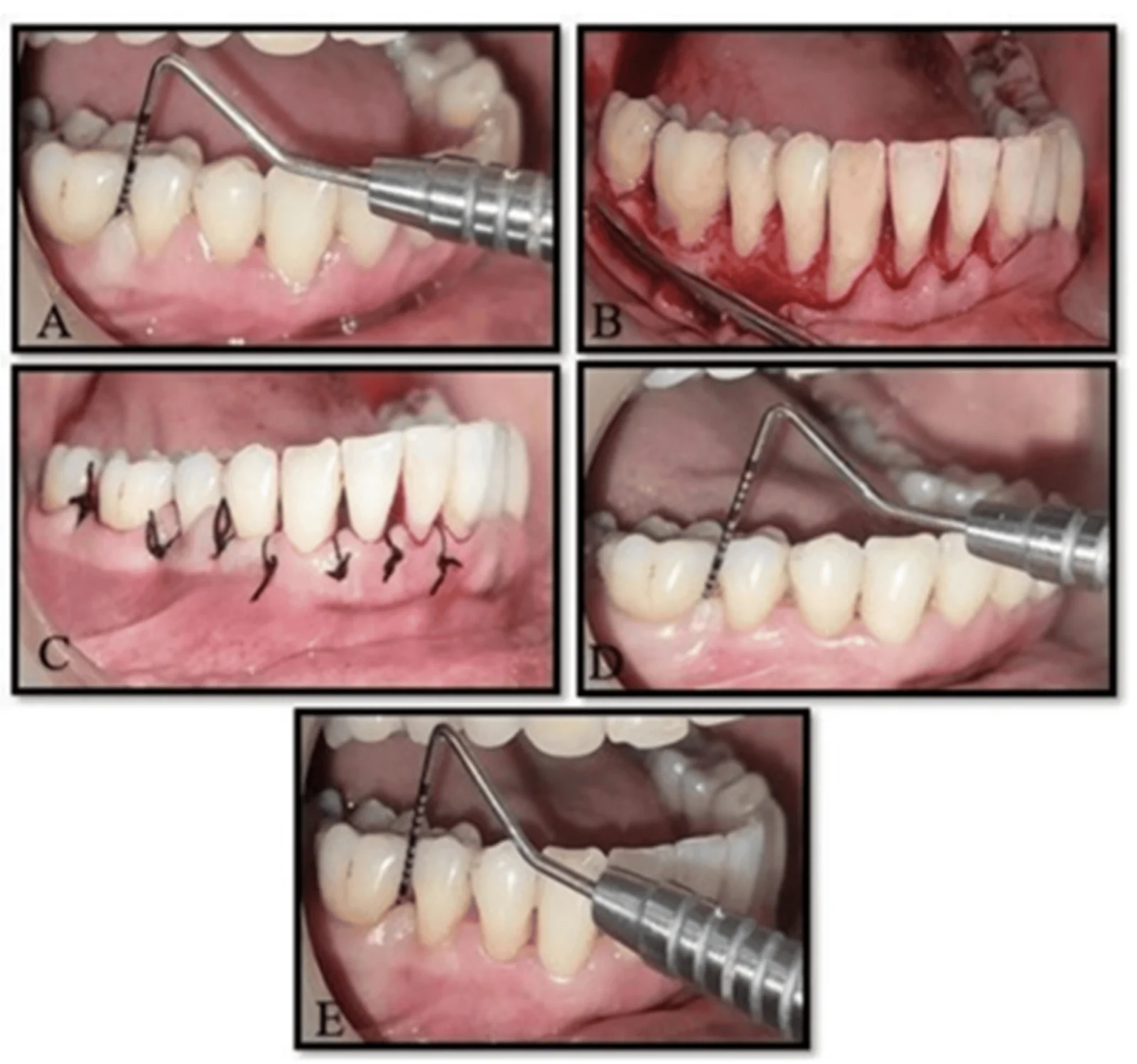
Clinical procedure and follow-up in Group A Figure shows Group A treated with open flap debridement alone: A, pre-surgical right lateral view; B, open flap debridement; C, suturing with 4-0 silk sutures; D, three-month follow-up right lateral view; E, six-month follow-up right lateral view.

Representative clinical images of the three treatment groups are presented to demonstrate the pre-surgical condition, surgical procedure, adjunctive intervention where applicable, suturing, and follow-up healing at three and six months. These figures contain representative clinical photographs and do not present numerical or statistical data; therefore, n (%), mean ± standard deviation, and p-values are not applicable. The clinical procedure and follow-up findings for Group A are shown in Figure 2.

**Figure 3:**
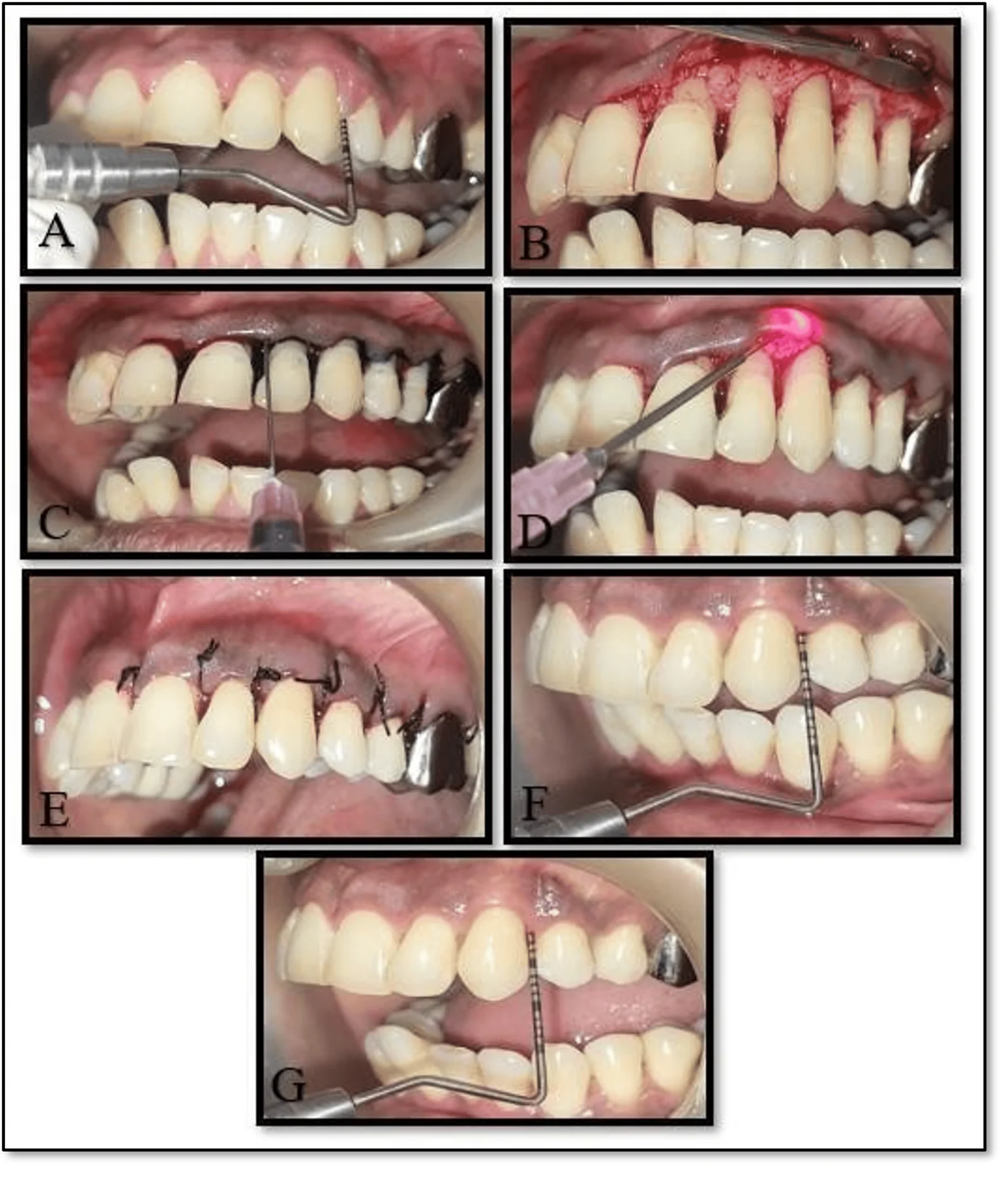
Clinical procedure and follow-up in Group B Figure shows Group B treated with open flap debridement and photodynamic therapy: A, pre-surgical left lateral view; B, open flap debridement; C, copious irrigation with methylene blue dye in the flap; D, application of 650 nm diode laser on the inner surface of the flap; E, suturing with 4-0 silk sutures; F, three-month follow-up right lateral view; G, six-month follow-up right lateral view.

Group B clinical procedure and follow-up are shown in Figure 3.

**Figure 4:**
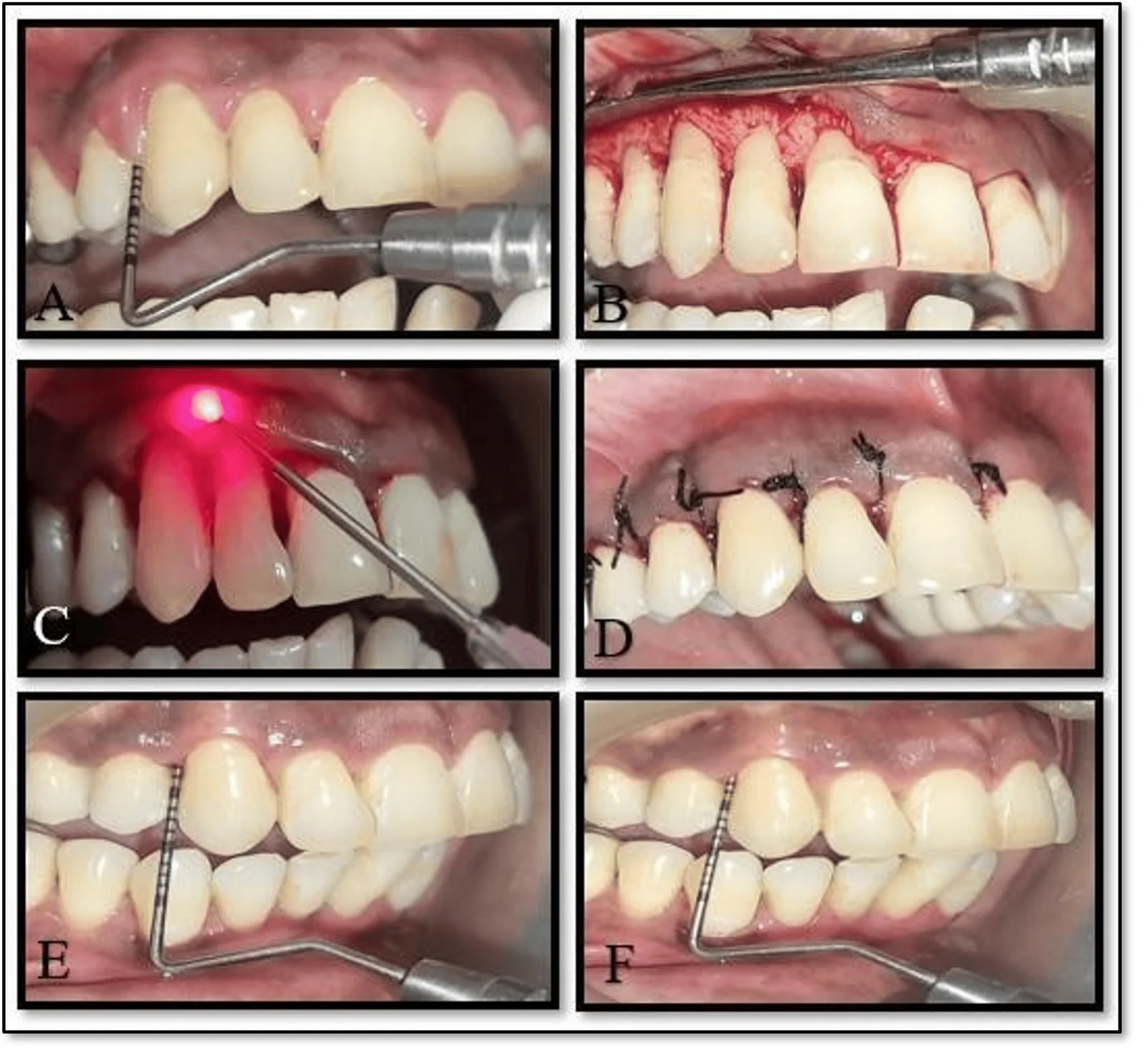
Clinical procedure and follow-up in Group C Figure shows Group C treated with open flap debridement and photoablation therapy: A, pre-surgical right lateral view; B, open flap debridement; C, photoablation using 980 nm diode laser on the inner surface of the flap; D, suturing with 4-0 silk sutures; E, three-month follow-up right lateral view; F, six-month follow-up right lateral view.

Group C clinical procedure and follow-up are shown in Figure 4.

## Discussion

This randomised controlled trial assessed the clinical effects of adjunctive PDT and photoablation in patients with periodontitis undergoing open flap debridement. The final analysis included 45 patients, with 15 patients in each treatment group. Significant improvements in clinical periodontal and bleeding-related parameters were observed from baseline to follow-up in all three groups. The laser-assisted groups demonstrated additional benefits, particularly in Clinical Attachment Level, Gingival Recession, and Papillary Bleeding Index at six months. These findings suggest that PDT and photoablation may improve selected clinical healing and inflammatory-control outcomes when used as adjuncts to conventional open flap debridement.

The enhanced outcomes observed with PDT may be related to its antimicrobial and anti-inflammatory properties. Randomised controlled studies have demonstrated beneficial clinical and microbiological outcomes following indocyanine green-based antimicrobial PDT combined with non-surgical periodontal treatment in patients with periodontitis [11]. In the present study, methylene blue was used as the photosensitising agent with a 650 nm diode laser, although the underlying therapeutic principle was similar. Activation of an appropriate photosensitiser by light generates reactive oxygen species capable of damaging bacterial cells and reducing the local microbial burden. Although microbiological changes were not measured in this trial, this mechanism may partly explain the reduced bleeding scores and greater clinical attachment improvement observed in the PDT group.

Clinical evidence has also demonstrated microbiological and immunological effects of antimicrobial PDT in recurrent and persistent periodontal pockets [12]. This evidence is relevant because the enrolled patients had residual probing pocket depths after Phase I therapy. Such sites may retain microorganisms in areas that are difficult to access through mechanical instrumentation alone. PDT applied after open flap debridement may therefore provide additional local decontamination under direct surgical access, although this proposed mechanism was not directly evaluated in the present study.

Laser-assisted treatment has also shown benefits in peri-implant and inflammatory periodontal diseases. A combined neodymium-doped yttrium aluminium garnet and erbium-doped yttrium aluminium garnet laser protocol has been reported to influence clinical and biomarker outcomes during the surgical treatment of peri-implantitis [13]. Although peri-implantitis differs from periodontitis, these findings support the potential role of laser-assisted decontamination and tissue modulation in inflammatory oral diseases. Combined Er:YAG and Nd:YAG laser therapy has also produced short-term clinical improvements in periodontitis [14].

The photoablation findings are consistent with evidence concerning diode laser-assisted periodontal surgery. A 445 nm diode laser has demonstrated clinical benefits as an adjunct to Kirkland flap surgery in periodontitis [15]. Diode laser-assisted surgical periodontal therapy has also demonstrated clinical and bacteriological effectiveness in a randomised split-mouth trial [16]. In the present study, the photoablation group showed greater clinical attachment gain and less gingival recession than the open flap debridement group. These benefits may be associated with the removal of inflamed pocket epithelium, reduction of bacterial reservoirs, improved soft-tissue adaptation, and enhanced wound healing; however, these mechanisms remain inferential because microbiological and histological assessments were not performed.

Diode laser therapy used as an adjunct to open flap debridement has also been associated with improved periodontal outcomes [17]. The effects of diode laser exposure during conventional flap surgery may extend beyond standard clinical periodontal parameters to postoperative pain and tissue response [18]. A systematic review and meta-analysis reported that diode laser-assisted flap surgery may improve pain control and clinical outcomes in chronic periodontitis [19]. The present study found no significant difference between the PDT and photoablation groups, suggesting comparable short-term clinical effectiveness. These findings support the potential use of either approach with open flap debridement, although larger trials with longer follow-up, microbiological assessment, patient-reported outcomes, and standardised laser parameters are required to establish long-term predictability.

Limitations and future directions

The present study has several limitations that should be considered when interpreting the findings. The final sample comprised 45 patients, with 15 patients in each group, which may have limited statistical power and the generalisability of the results to populations with different disease severity, oral-hygiene status, and risk-factor profiles. The six-month follow-up period may not be sufficient to assess the long-term stability of Clinical Attachment Level, Gingival Recession, and Papillary Bleeding Index outcomes. Although the principal surgical procedures and laser parameters were reported, device-specific information and postoperative management protocols were not documented comprehensively, which may limit exact replication across clinical settings. The absence of microbiological and inflammatory biomarker analyses restricted mechanistic interpretation because changes in periodontal pathogen burden and host inflammatory responses were not directly assessed. Patient-reported outcomes, including postoperative pain, discomfort, satisfaction, and oral health-related quality of life, were also not evaluated. The absence of formal examiner calibration and quantitative intra-examiner reliability assessment may have introduced measurement variability.

Future multicentre studies with larger, adequately powered samples and longer follow-up are required to establish the stability, durability, and predictability of PDT and photoablation. Standardisation of laser parameters, photosensitiser protocols, irradiation times, device characteristics, postoperative care, and reporting methods would improve reproducibility and comparability. Future investigations should incorporate microbiological profiling, inflammatory biomarkers, radiographic assessment, examiner-reliability testing, and patient-centred outcomes to provide a more comprehensive evaluation of therapeutic efficacy and clinical relevance.

## Conclusions

Both PDT and photoablation, used as adjuncts to open flap debridement, produced greater improvement in clinical attachment level, gingival recession, and papillary bleeding index than open flap debridement alone at six months. The results suggest a potential clinical benefit of adjunctive laser-assisted therapy in patients with periodontitis, particularly for periodontal healing and inflammatory control following surgical debridement. Both PDT and photoablation demonstrated similar efficacy, indicating that both techniques may be considered potential adjunctive options for residual periodontal pockets after Phase I therapy. The findings should be interpreted cautiously because of the limited sample size and six-month follow-up period. Larger, long-term randomised controlled trials are required to confirm the stability, predictability, and applicability of these techniques in routine clinical practice.
